# Association of survival with adjuvant radiotherapy for pN0 esophageal cancer

**DOI:** 10.18632/aging.204677

**Published:** 2023-04-25

**Authors:** Huijiang Gao, Yuanyong Wang, Zhihui Jiang, Guodong Shi, Shiyu Hu, Jiangshan Ai, Zhaofeng Wang, Yucheng Wei

**Affiliations:** 1 Department of Thoracic Surgery, The Affiliated Hospital of Qingdao University, Qingdao, China; 2 Department of Thoracic Surgery, Tangdu Hospital of Air Force Military Medical University, Xi’an, China; 3 Department of General Surgery, Qingdao Women and Children’s Hospital, Qingdao, China

**Keywords:** esophageal cancer, adjuvant radiotherapy, surgery, SEER, survival

## Abstract

Introduction: This study was conducted to elucidate the link between adjuvant radiotherapy and survival in pathologic node-negative (pN0) esophageal cancer patients with upfront esophagectomy.

Methods: From 2000 to 2016, patients with pN0 esophageal cancer who underwent upfront esophagectomy were selected from the Surveillance, Epidemiology, and End Results (SEER) database. The association of high-risk covariates with survival after adjuvant radiotherapy was evaluated using propensity score matching and multivariate analysis.

Results: We identified 3197 patients, 321 (10.0%) underwent postoperative radiotherapy and 2876 (90.0%) underwent esophagectomy alone. In the unmatched cohort, postoperative radiotherapy was associated with a statistically significant but modest absolute decrease in survival outcomes (P < 0.001). In the matched cohort, the survival differences disappeared. Additionally, adjuvant radiotherapy was linked to a 5-year overall survival (OS) benefit for patients with the pT3-4N0 disease (34.8% vs. 27.7%; P = 0.008). Adjuvant radiotherapy for pT3-4N0 disease with tumor length ≥3 cm, adenocarcinoma, and evaluated lymph node count <12 was shown to independently function as a risk factor for improved OS, as per a multivariate analysis (P < 0.01).

Conclusions: This population-based trial showed that high-risk patients with pT3-4N0 esophageal cancer had better OS following upfront esophagectomy followed by radiotherapy therapy. This discovery may have major significance in the use of adjuvant radiotherapy following upfront esophagectomy in patients with pN0 esophageal cancer.

## INTRODUCTION

Esophageal cancer may be treated with a variety of approaches, including neoadjuvant chemoradiotherapy (chemoRT), upfront esophagectomy, and definitive chemoradiotherapy [1]. Patients with diagnosed locoregional illness are recommended to receive a comprehensive treatment plan, as per national recommendations, while those with locally advanced esophageal cancer could be ideal candidates for an upfront esophagectomy [2–5]. Currently, it is still unclear what kind of postoperative care is best for individuals who receive upfront surgical resection.

Multiple studies have been conducted to investigate the risk factors that are linked to esophageal cancer survival following radical resection [6–9]. Adjuvant treatment, which may enhance prognosis by decreasing the risk of cancer recurrence and subsequently removing remaining tumors and metastatic lymph nodes, is considered among the most significant factors that determine the prognostic outcomes [10–14]. Multiple studies have been conducted to investigate the use of adjuvant radiotherapy, however, the outcomes of these research have been inconsistent for individuals who have undergone upfront esophagectomy [15–17]. In addition, adjuvant chemotherapy has been the subject of various randomized clinical studies, none of which have shown that it improves patients’ chances of survival [18]. For patients with locally advanced (T2-T4N0-1) adenocarcinoma or non-radical resection, the NCCN guidelines presently recommend postoperative chemoradiotherapy despite the absence of high-level evidence [1].

We conducted a retrospective analysis of the Surveillance, Epidemiology, and End Results (SEER) database to identify risk factors for receiving adjuvant radiotherapy following upfront esophagectomy for pathologic node-negative (pN0) esophageal cancer and assess the effect of this treatment on survival.

## MATERIALS AND METHODS

### Patient selection

The SEER database is a comprehensive data resource that includes the data of nearly 30% of the US population from 18 registries across the US [19]. Data from the SEER database were used to retrospectively compile patient demographics, disease features, treatment options, and outcomes. This population-based tumor registry only collected data on patients with esophageal cancer from 2000 to 2016 (*n* = 64625). OS and CSS, defined as the date of the initial diagnosis to the event of all-cause or cancer-related mortality, respectively, were the primary outcomes of this research.

**Figure 1:**
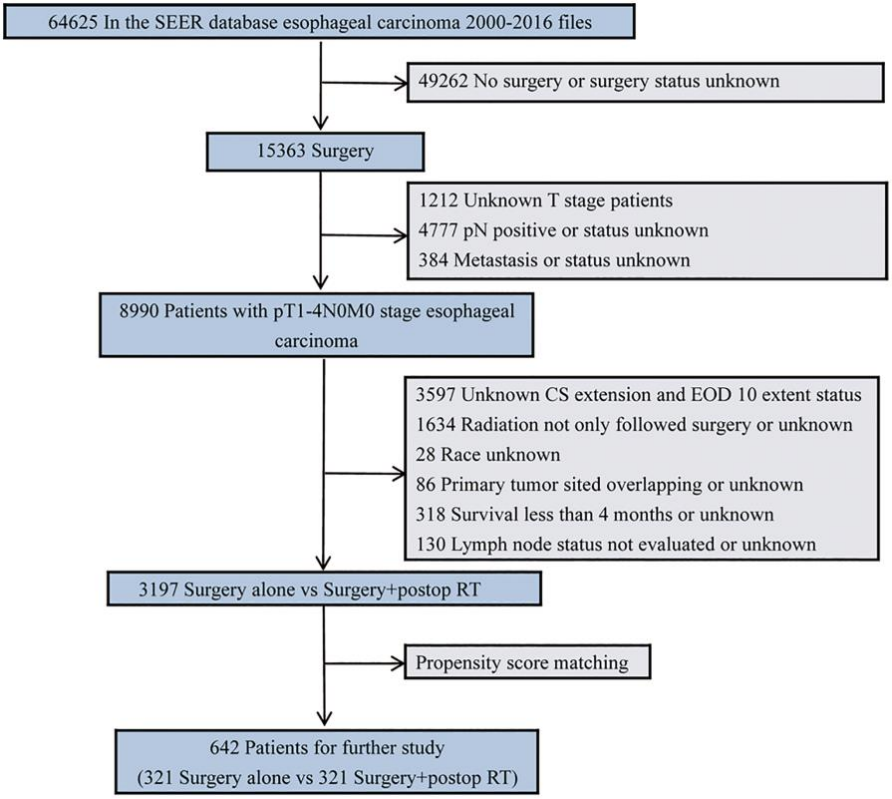
Diagram of the primary study cohort selection steps.

From 2000 to 2016, patients diagnosed with pN0 esophageal cancer and undergoing upfront esophagectomy were selected for this study. Patient selection criteria are summarized in Figure 1. Only those who received upfront esophagectomy alone or postoperative radiotherapy (postop RT) were included for analysis. Cases were excluded if they were confirmed with pathologically diagnosed positive lymph nodes, had missing or incomplete data, or if they were <18 years old. Total radiotherapy dosage was not restricted to include patients undergoing a variety of radiotherapy protocols [20]. There was no need to seek approval from the Institutional Review Board since the data from SEER were deidentified. Besides, those with a median survival time of < 4 months were not included to mitigate any bias in favor of adjuvant radiotherapy, given that some patients may wait four months followed by adjuvant radiotherapy after receiving the operation [21].

### Statistical methods

SPSS (version 23.0; IBM, Armonk, NY, USA) was utilized for the analyses of statistical data. For continuous variables, the mean and standard deviation were employed to demonstrate the normality of distribution, while the percentage was employed for discrete data. Patient groups were matched using the propensity score, which was developed to eliminate baseline demographic data and improve grouping homogeneity [22]. Patients who had surgery alone (SA) or surgery followed by postoperative radiotherapy (S+RT) were matched 1: 1 using a caliper width cutoff of 0.1-fold of the SD (Supplementary Figures 1, 2). Kaplan-Meier (KM) analysis was conducted to produce OS and CSS distributions, which were then analyzed with the log-rank test. Propensity score matching (PSM), along with subsequent multivariate Cox proportional hazards regression analysis was conducted and two-sided *P*-values of < 0.05 were defined as the significance criterion.

### Data availability statement

This study was based on the Surveillance, Epidemiology, and End Results (SEER) database. The SEER database is an open-accessed registry system collecting data from 18 registries among 14 states across the US, representing nearly 30% of the US population, and the data in our study is available on https://seer.cancer.gov/ through the online application.

## RESULTS

In total, we enrolled 3197 patients, of whom 321 (10.0%) received postop RT following surgery and 2876 (90.0%) received surgery alone. Table 1 lists the patient baseline characteristics by treatment groups (SA vs. S+RT). The findings illustrated that patients who received S+RT had significantly larger total tumor sizes as well as more localized and squamous cell carcinoma disease than those who received SA, all of which likely contributed to the poorer survival rates.

**Table 1:** Demographic and clinical characteristics of the study cohort.

**Characteristics**	**Surgery Alone**	**Surgery + postop RT**	***P*-value**
	**(*n* = 2876)**	**(*n* = 321)**	
Age, y, *n* (%)			0.036
<60	716 (24.9)	100 (31.2)	
60–70	971 (33.8)	106 (33.0)	
≥70	1189 (41.3)	115 (35.8)	
Male sex, *n* (%)	2287 (79.5)	251 (78.2)	0.577
Race/ethnicity, *n* (%)			0.134
White	2586 (89.9)	280 (87.2)	
Other	290 (10.1)	41 (12.8)	
Disease site, *n* (%)			0.046
Upper third	140 (4.9)	25 (7.8)	
Middle third	565 (19.6)	69 (21.5)	
Lower third	2171 (75.5)	227 (70.7)	
Tumor length, cm, *n* (%)			<0.001
<3	1815 (63.1)	123 (38.3)	
3–5	629 (21.9)	97 (30.2)	
≥5	432 (15.0)	101 (31.5)	
Tumor histology, *n* (%)			<0.001
Squamous cell carcinoma	695 (24.2)	110 (34.3)	
Adenocarcinoma	2181 (75.8)	211 (65.7)	
Histologic grade, *n* (%)			<0.001
Well + Moderate	2070 (72.0)	189 (58.9)	
Poor + Undifferentiated	806 (28.0)	132 (41.1)	
Pathological T stage, *n* (%)			<0.001
T1-2	2324 (80.8)	157 (48.9)	
T3-4	552 (19.2)	164 (51.1)	
ELN count, *n* (%)			0.143
<12	1777 (61.8)	216 (67.3)	
12–16	364 (12.7)	37 (11.5)	
≥16	735 (25.6)	68 (21.2)	

Abbreviations: postop RT: postoperative radiation therapy; ELN: examined lymph node.

**Figure 2:**
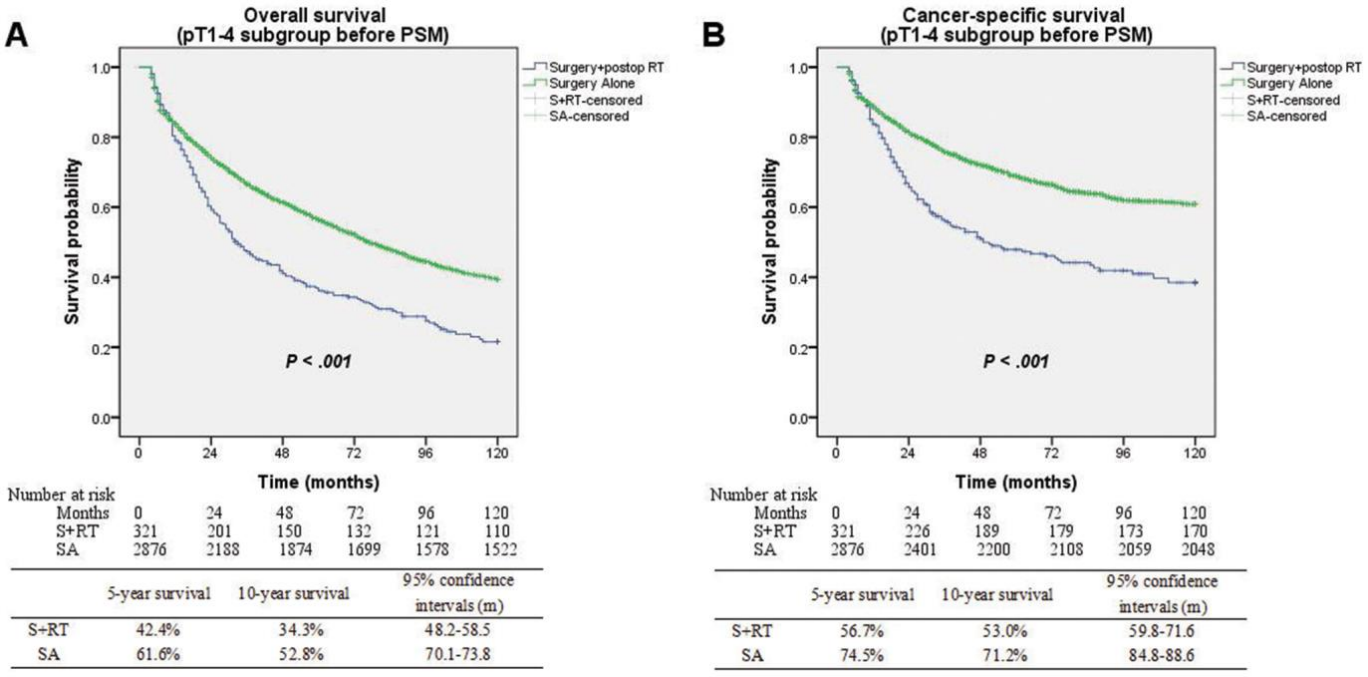
(**A**) Overall survival between surgery alone and surgery + postop RT groups before matching (*P* < 0.001). (**B**) Cancer-specific survival between surgery alone and surgery + postop RT groups before matching (*P* < 0.001).

**Figure 3:**
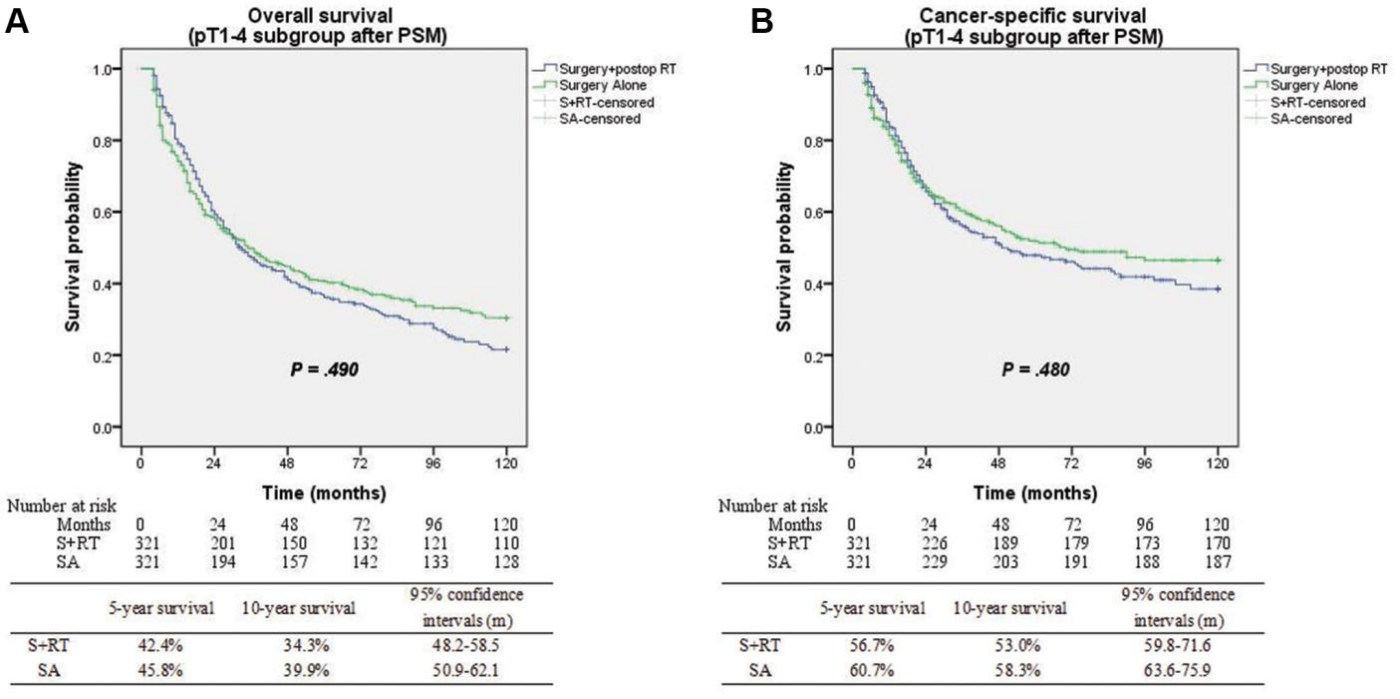
(**A**) Overall survival between surgery alone and surgery + postop RT groups after matching (*p* = 0.49). (**B**) Cancer-specific survival between surgery alone and surgery + postop RT groups after matching (*p* = 0.48).

Patients had a median follow-up duration of 50.5 months following diagnosis (95% CI: 49.1–51.9 months). Substantially improved OS (5-yr OS 61.6% vs. 42.4%; *P* < 0.001) and CSS (5-yr CSS 74.5% vs. 56.7%; *P* < 0.001) were seen among patients who underwent the SA treatment in contrast with those in the S+RT group (Figure 2). Matching and comparing 321 patients in the SA cohort to the same number of patients in the S+RT group were performed following PSM. The demographic variables without significant differences were considered (Table 2). When all of the patients who were matched were considered, the differences between the two groups in terms of OS (5-yr OS 45.8% vs. 42.4%; *P* = 0.893) and CSS (5-yr CSS 60.7% vs. 56.7%; *P* = 0.697) were insignificant (Figure 3).

**Table 2:** Demographic and clinical characteristics for pathologic node-negative patients after PSM.

**Characteristics**	**Surgery alone**	**Surgery + postop RT**	**Standardized difference**	
	**(*n* = 321)**	**(*n* = 321)**	**before after**	
Age, y, *n* (%)			0.166	0.032
<60	94 (29.3)	100 (31.2)		
60–70	107 (33.3)	106 (33.0)		
≥70	120 (37.4)	115 (35.8)		
Male sex, *n* (%)	239 (74.5)	251 (78.2)	0.033	−0.093
Race/ethnicity, *n* (%)			−0.089	0.083
White	272 (84.7)	280 (87.2)		
Other	49 (15.3)	41 (12.8)		
Disease site, *n* (%)			0.139	0.017
Upper third	25 (7.8)	25 (7.8)		
Middle third	66 (20.6)	69 (21.5)		
Lower third	230 (71.7)	227 (70.7)		
Tumor length, cm, *n* (%)			−0.556	0.076
<3	110 (34.3)	123 (38.3)		
3–5	105 (32.7)	97 (30.2)		
≥5	106 (33.0)	101 (31.5)		
Tumor histology, *n* (%)			0.236	−0.116
Squamous cell carcinoma	126 (39.3)	110 (34.3)		
Adenocarcinoma	195 (60.7)	211 (65.7)		
Histologic grade, *n* (%)			−0.292	−0.076
Well + Moderate	200 (62.3)	189 (58.9)		
Poor + Undifferentiated	121 (37.7)	132 (41.1)		
Pathological T stage, *n* (%)			−0.810	−0.04
T1-2	162 (50.5)	157 (48.9)		
T3-4	159 (49.5)	164 (51.1)		
ELN count, *n* (%)			0.115	−0.04
<12	220 (68.5)	216 (67.3)		
12–16	40 (12.5)	37 (11.5)		
≥16	61 (19.0)	68 (21.2)		

Abbreviations: postop RT: postoperative radiotherapy; ELN: examined lymph node.

**Figure 4:**
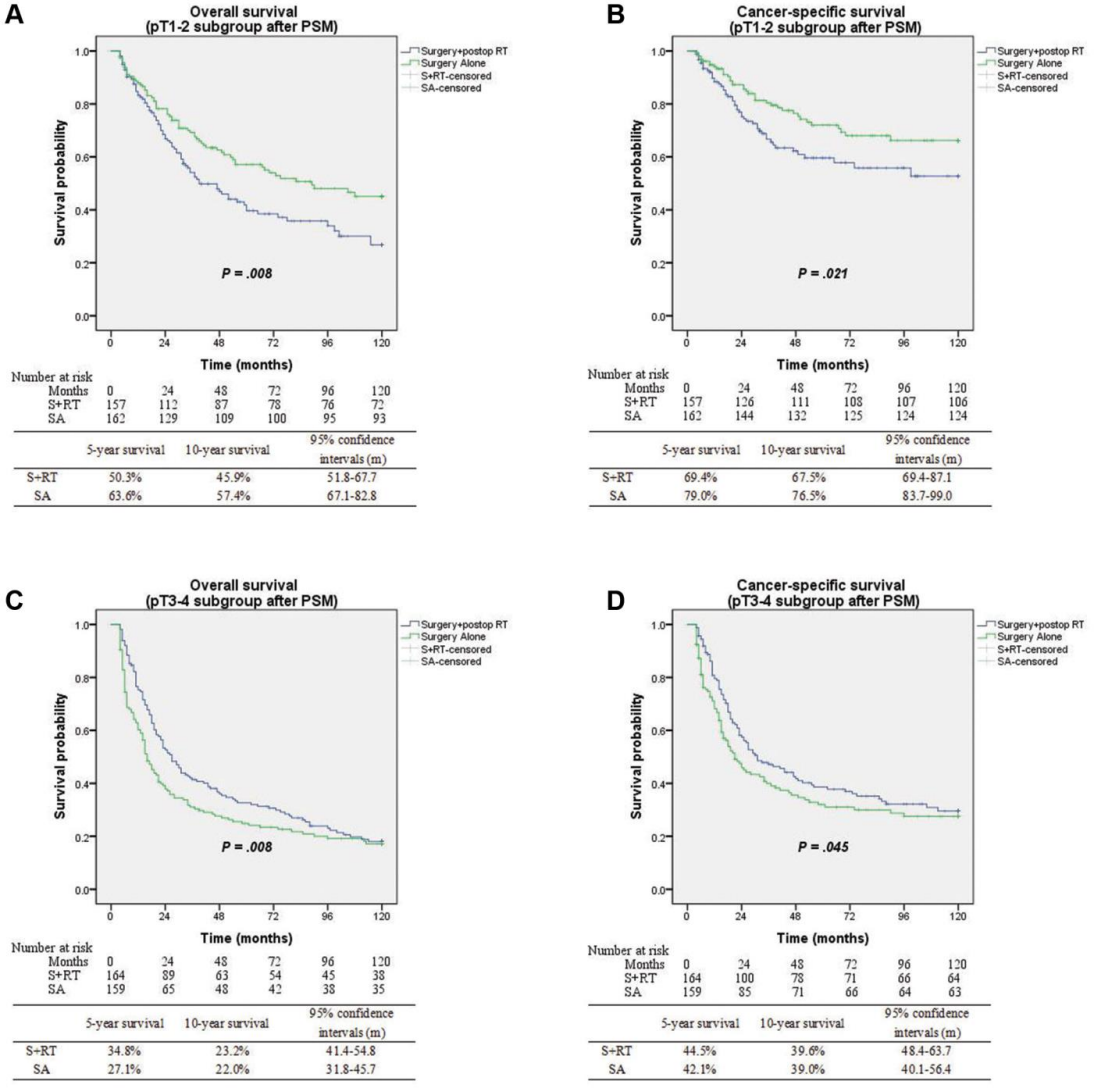
(**A**) Overall survival between surgery alone and surgery + postop RT groups with pT1-2 subgroup (*p* = 0.008). (**B**) Cancer-specific survival between surgery alone and surgery + postop RT groups with pT1-2 subgroup (*p* = 0.021). (**C**) Overall survival between surgery alone and surgery + postop RT groups with pT3-4 subgroup (*p* = 0.008). (**D**) Cancer-specific survival between surgery alone and surgery + postop RT groups with pT3-4 subgroup (*p* = 0.045).

On subgroup analysis incorporating pathological tumor stages, postoperative radiotherapy was associated with improved OS (5-yr survival 34.8% vs. 27.7%; *P* = 0.008) and CSS (5-yr survival 44.5% vs. 42.1%; *P* = 0.045) for pT3-4N0 disease. However, a significant but modest absolute decline in overall and cancer-specific survival outcomes for patients diagnosed with pT1-2N0 (early-stage) disease (*P* = 0.008 and *P* = 0.021, respectively) (Figure 4). Based on the findings of multivariate analysis, postop RT was linked to favorable OS, with an HR value of 0.69; 95% CI, 0.52–0.89; and *P* = 0.006 for pT3-4N0 disease, but with a decreased OS for early-stage disease (HR 1.43; 95% CI, 1.02–2.02; *P* = 0.04). Additionally, the multivariate analysis of pT3-4N0 disease illustrated that adjuvant radiotherapy for tumor length ≥3 cm (*P* = 0.002; 95% CI, 0.48–0.85), adenocarcinoma (*P* = 0.006; 95% CI, 0.36–0.84), and ELN count <12 (*P* = 0.003; 95% CI, 0.43–0.84) was a powerful prognostic factor linked to favorable OS relative to surgery alone procedure in Table 3.

**Table 3:** Univariable and multivariable HRs for overall survival according to pT3-4 subgroup characteristics after PSM.

**Cohort**	**Surgery alone**	**Surgery + postop RT**	**Univariable analysis**		**Multivariable analysis**	
	**(*n* = 159)**	**(*n* = 164)**	**HR (95% CI)**	***P* value**	**HR (95% CI)**	***P* value**
Age, *n* (%)						
<60	50 (31.4)	62 (37.8)	0.54 (0.35–0.84)	0.006	0.57 (0.35–0.94)	0.026
60–70	48 (30.2)	54 (32.9)	1.08 (0.66–1.78)	0.762	1.31 (0.74–2.32)	0.352
≥70	61 (38.4)	48 (29.3)	0.67 (0.43–1.05)	0.081	0.58 (0.36–0.94)	0.056
Sex, *n* (%)						
Male	107 (67.3)	125 (76.2)	0.74 (0.55–1.01)	0.061	0.72 (0.51–1.00)	0.050
Female	52 (32.7)	39 (23.8)	0.61 (0.37–1.02)	0.061	0.60 (0.35–1.03)	0.062
Race/ethnicity, *n* (%)						
White	127 (79.9)	137 (83.5)	0.68 (0.51–0.92)	0.011	0.66 (0.48–0.91)	0.010
Other	32 (20.1)	27 (16.5)	0.85 (0.47–1.53)	0.585	0.77 (0.39–1.52)	0.453
Disease site, *n* (%)						
Upper third	20 (12.6)	16 (9.8)	0.52 (0.25–1.09)	0.085	0.48 (0.15–1.47)	0.197
Middle third	44 (27.7)	44 (26.8)	0.84 (0.52–1.34)	0.464	0.88 (0.54–1.46)	0.629
Lower third	95 (59.7)	104 (63.4)	0.68 (0.48–0.97)	0.035	0.69 (0.48–0.99)	0.046
Tumor length, *n* (%)						
<3 cm	21 (13.2)	26 (15.9)	1.02 (0.47–2.21)	0.955	1.17 (0.46–3.00)	0.741
≥3 cm	138 (86.8)	138 (84.1)	0.67 (0.51–0.89)	0.005	0.64 (0.48–0.85)	0.002
Histology, *n* (%)						
SCC	93 (58.5)	72 (43.9)	0.93 (0.66–1.32)	0.689	0.83 (0.58–1.21)	0.336
Adenocarcinoma	66 (41.5)	92 (56.1)	0.57 (0.38–0.86)	0.007	0.55 (0.36–0.84)	0.006
Histologic grade, *n* (%)						
Well + Moderate	87 (54.7)	91 (55.5)	0.75 (0.52–1.08)	0.124	0.84 (0.57–1.23)	0.364
Poor + Undifferentiated	72 (45.3)	73 (44.5)	0.66 (0.45–0.96)	0.031	0.60 (0.39–0.90)	0.015
ELN count, *n* (%)						
<12	113 (71.1)	93 (56.7)	0.69 (0.49–0.95)	0.024	0.60 (0.43–0.84)	0.003
12–16	19 (11.9)	23 (14.0)	0.72 (0.31–1.67)	0.450	0.31 (0.09–1.11)	0.071
≥16	27 (17.0)	48 (29.3)	0.92 (0.51–1.65)	0.774	0.96 (0.50–1.83)	0.896

Abbreviations: postop RT: postoperative radiotherapy; SCC: squamous cell carcinoma; ELN: examined lymph node.

## DISCUSSION

According to the current data on esophageal cancer, patients with locally advanced diseases who are candidates for surgical resection ought to be treated with either neoadjuvant chemoradiotherapy or induction chemotherapy. Despite this, a number of patients with clinically identified locoregional illnesses received upfront esophagectomies [23–25]. Postoperative adjuvant radiotherapy has been demonstrated to improve the survival of pathologic node-positive patients with upfront esophagectomies in several clinical trials. Despite the prevalence of this clinical condition, there is a paucity of data on whether or not pN0 patients who have already had upfront surgery benefit from subsequent adjuvant radiotherapy [15–17].

Currently, the optimal treatment procedure is still uncertain for pN0 esophageal cancer following an upfront esophagectomy. The aforementioned studies proposed surveillance for pN0 patients receiving esophagectomy alone. However, it is plausible that those studies did not have enough power to accurately stratify the results based on the state of the nodes. For example, Wong and colleagues retrospectively analyzed 4893 patients from the National Cancer Database including esophageal cancer patients in pT3-4Nx-0 or pT1-4N1-3 stage without metastatic disease, and 1153 of these patients had postoperative radiotherapy and exhibited a statistically significant but moderate absolute improvement in survival relative to those who just underwent surgery (HR 0.77, 95% CI, 0.71–0.83; *P* < 0.001). However, when establishing subgroups based on the state of their lymph nodes, patients who did not have cancer in their lymph nodes seemed to gain no substantial benefit from postoperative radiotherapy in terms of their survival rates [15]. Furthermore, using data from SEER, Shridhar et al. conducted a retrospective research that included 2109 patients with esophageal cancer who had had esophagectomies followed by adjuvant radiotherapy. Of those patients, 1373 were classified as having negative lymph node status. They found that postop RT significantly decreased survival for node-negative patients compared to esophagectomy alone, but there were no survival differences between the pT stages [16]. Postoperative radiotherapy was linked to enhanced local control and could improve the OS for patients in pN0 and pN1 categories, as reported by Chen et al. in their single-institution cohort of 692 T3 esophageal squamous cell carcinoma patients who underwent radical resection [17]. Therefore, the survival analysis for those locoregional diseases has not been demonstrated to stratify the results by lymph node status. Additionally, there is a dearth of information on whether individuals with node-negative esophageal cancer following upfront esophagectomy gain from adjuvant treatment in terms of their survival.

Unique to those aforementioned retrospective studies, our study focused on the context of postop RT use only for patients with tumor-negative lymph node status. Compared to patients who did not receive SA, those who received SA exhibited considerably smaller overall tumor sizes, an early pT stage, a higher percentage of adenocarcinoma, and a highly differentiated histologic grade in this large hospital-based trial. We found that esophagectomy alone was associated with a 19.2% 5-year OS and 17.8% 5-year CSS benefit compared to esophagectomies followed by postop RT. Nonetheless, PSM did not significantly improve survival rates. Postoperative RT following esophagectomy was linked to a 7.7% absolute 5-year OS benefit for pT3-4N0 disease relative to the SA group in a subgroup analysis. This outcome was driven by patients whose tumors were ≥3 cm in length, were adenocarcinomas, and had ELN counts of <12, as determined by multivariate Cox regression subgroup analysis. The S+RT cohort was linked to a statistically significant but moderate absolute decline in survival status in patients with early-stage disease. Because unnecessary and potentially hazardous therapy might cause possible health deterioration, radiotherapy-related morbidity, or death in this group, surgery alone was preferred for individuals with early-stage disease less than pT3-4N0. Besides, this SEER-based analysis also found that identifying higher-risk factors was likely to offer a favorable prognosis than a uniform strategy for the pT3-4N0 disease after upfront esophagectomy.

In addition to the TNM staging system, tumor length is a specific variable for esophageal cancer which is reported to independently function as a prognostic marker in multiple research reports [23, 25, 26]. Semenkovich et al. constructed a decision analysis model and analyzed data from 10 trials involving 4013 patients with cT2N0 esophageal cancer. A tumor length >3 cm independently served as a risk indicator linked to a likelihood of upstaging risk of >48.1% [25]. Shridhar and colleagues reviewed 1840 esophageal cancer patients who underwent esophagectomy from 2004 to 2013 in the National Cancer Database. They discovered that factors such as poor differentiation and tumor lengths >3 centimeters were substantially linked to tumor upstaging. Drawing on data from the National Cancer Database, another research conducted a retrospective analysis of 735 matched T1a esophageal adenocarcinoma pairs of patients who had undergone esophagectomies or endoscopic resections, which also demonstrated that tumor length was one of the primary factors linked to nodal metastases [26]. Thus, these studies indicated that tumor length might be an independent risk factor associating survival benefits with induction and/or adjuvant therapy. As per the findings of our research, postoperative radiotherapy is not an independent risk indicator for survival as determined by multivariate analysis of all cohort patients. However, tumor length ≥3 cm was an independent predictor for improved OS with postoperative RT relative to patients who received only upfront surgery as shown by the multivariate analysis for all-cause mortality in the pT3-4N0 subgroup.

Advancements in endoscopic surveillance and detection have led to earlier diagnoses of esophageal cancer [27]. For more accurate tumor staging and improved diagnosis, surgery should include not only extensively resecting the lesion, but also dissecting the possibly metastatic nodes. In the past decades, numerous research publications have scrutinized the impact of the ELN count on survival for esophageal cancer patients, but the standard lymph node dissection for esophagectomy is still contentious [28–30]. A parallel cohort analysis utilizing the database of the Worldwide Esophageal Cancer Collaboration illustrated that the degree of lymphadenectomy was linked to survival rates for patients who had pT1N0 esophageal cancer and that the ideal number of ELN was 10 to 12 nodes [28]. In their study, Yu et al. conducted a retrospective analysis of 194 patients with pN0 ESCC who had undergone radical esophagectomies and found that the minimal number of ELN for pN0 ESCC should be 14 nodes [29]. Furthermore, fewer lymph nodes examined were correlated with a higher rate of regional failure, and the locoregional recurrence-free survival was longer in patients who had >13 lymph nodes evaluated compared to patients with ≤13 ELNs, as per the research by Shaikh et al. [30]. The findings of our study were in line with those of other investigations done in the past. We identified a correlation between the condition (if less than 12 lymph nodes were removed following postoperative radiotherapy) and enhanced survival for patients with stages pT3-4N0 cancer. This might be due to stage migration since the risk of discovering occult nodal metastases rises with the number of lymph nodes that are removed during the surgical procedure.

This is the largest research that we are aware of to date that particularly examines the effect of postoperative radiotherapy treatment for patients with pN0 who have undergone upfront esophagectomy. However, this population-based study is not without its drawbacks. To begin, we conducted an observational study to assess our data. Patients were divided into treatment groups (SA versus SA+RT), however, they were not chosen at random for either group, which might have led to selection bias. In addition, data regarding chemotherapy administration was lacking. Many of the patients who received radiotherapy also had chemotherapy. However, it was unclear how much chemotherapy dosage was administered or whether the dosage was lowered at any point throughout treatment. Additional research is needed on this topic since chemotherapy protocols have changed dramatically over the past several decades, and survival outcomes are dependent upon the chemotherapy used. Furthermore, another inherent limitation of the SEER database was the amount of missing data. Within the cohort of patients with survival data, there is a substantial proportion (21.2%) of patients with missing data on demographics or tumor characteristics. Although restricting our analysis to those patients with complete data resulted in a smaller sample size, it provided for a more stringent comparison. Finally, it is difficult to ascertain if the survival benefits could be related to unmeasured confounding variables including patient comorbid conditions, functional status, lymphovascular infiltration, type of lymphadenectomy, and toxicological data, which are not included in the SEER.

## CONCLUSIONS

This large population-based study showed that patients with pT3-4N0 esophageal cancer who underwent upfront esophagectomy benefited from receiving postoperative radiotherapy in terms of OS, particularly for individuals with adenocarcinoma tumors ≥3 cm in length and examined number of lymph node <12. This discovery may have major value for pN0 esophageal cancer patients who have received upfront surgery followed by adjuvant radiotherapy.

## Supplementary Materials

Supplementary Figures
