# Supplementary material for: Association of survival with adjuvant radiotherapy for pN0 esophageal cancer
Source: Aging (Albany NY). 2023 Apr 25;15(8):3158–70. doi: 10.18632/aging.204677 (PMC10188340; doi:10.18632/aging.204677)
Supplement: Supplementary Figures [file aging-15-204677-s001.pdf]

SUPPLEMENTARY FIGURES

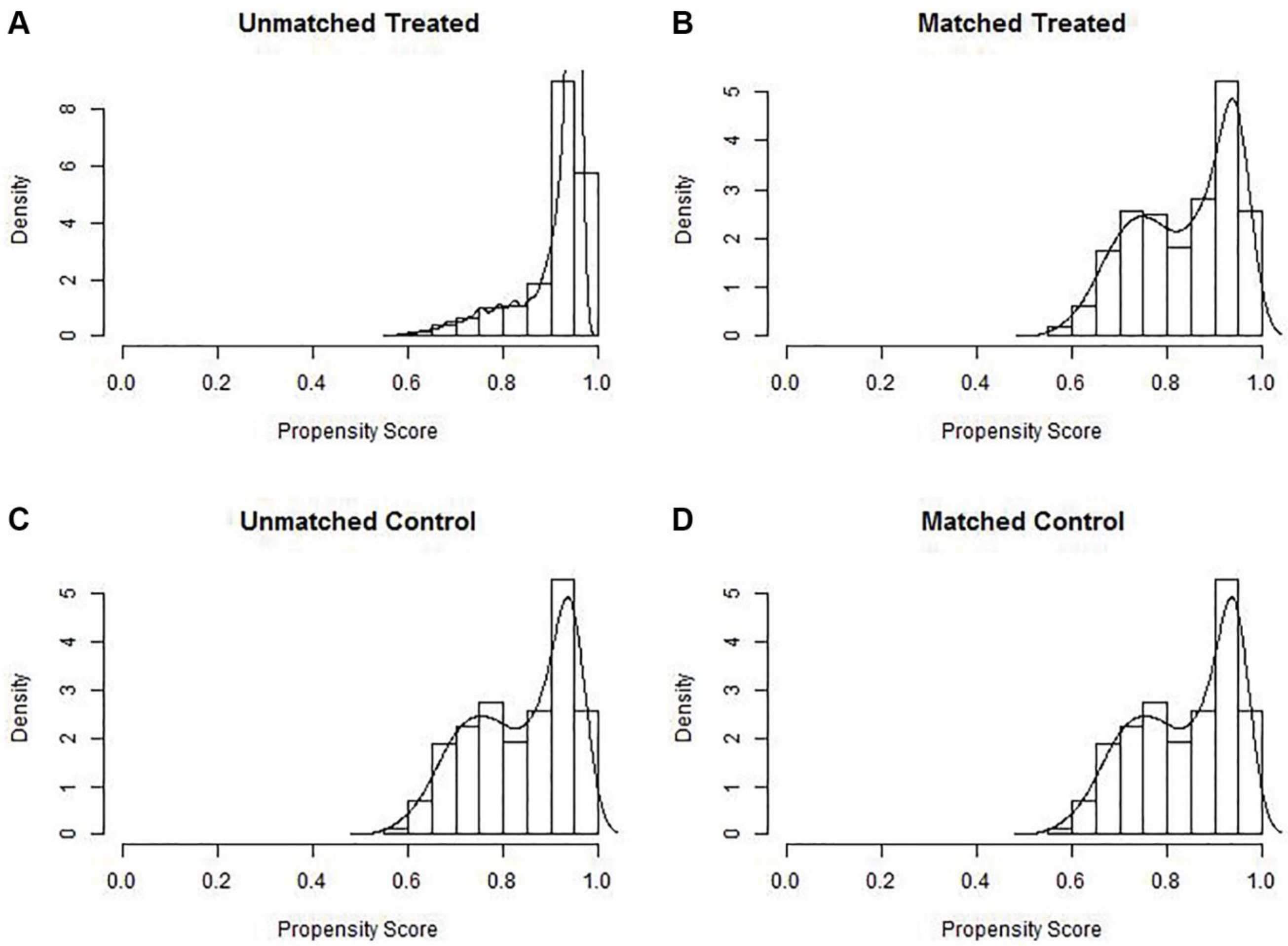

Supplementary Figure 1. Histogram of propensity scores for patients between the surgery alone group and surgery + postop RT group. (**A**) Unmatched patients who received surgery alone. (**B**) Matched patients who received surgery alone. (**C**) Unmatched patients who received surgery + postop RT. (**D**) Matched patients who received surgery + postop RT. Matched groups have similar propensity score distributions.

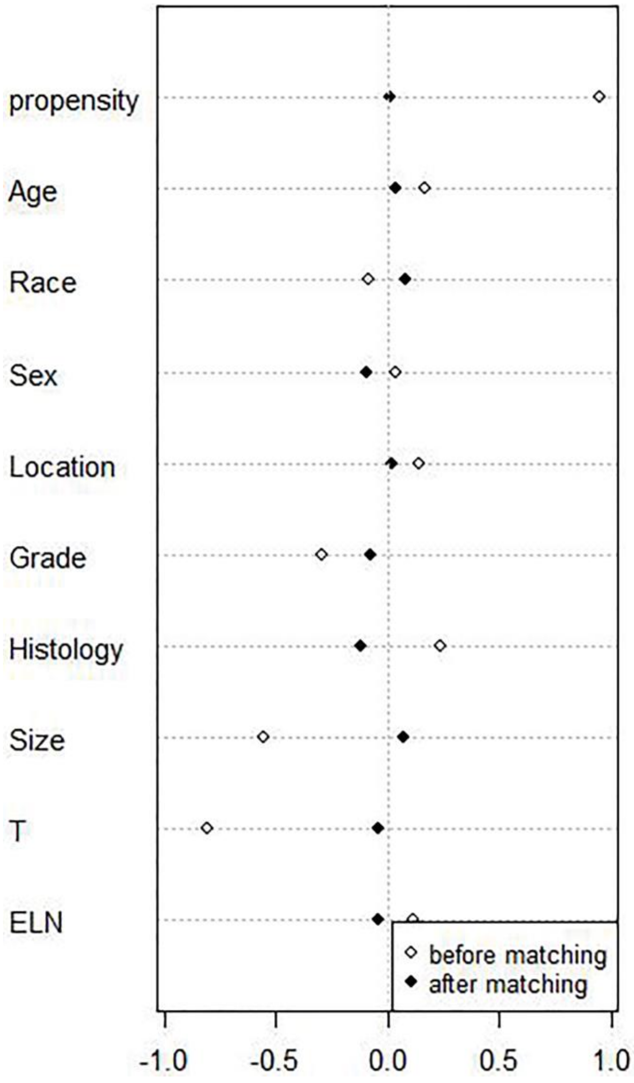

Supplementary Figure 2. Standardized differences of variables between patients who received surgery alone and those who received surgery + postop RT. Hollow diamond symbolized differences before propensity matching and black diamond symbolized differences after propensity matching. Propensity matching effectively reduced heterogeneity among variables between the two surgical approaches in comparison. Abbreviation: ELN: examined lymph node.
